# Subcutaneous Leiomyosarcoma of the Frenulum

**DOI:** 10.1100/tsw.2005.76

**Published:** 2005-08-01

**Authors:** D. Mendis, S. R. J. Bott, J. H. Davies

**Affiliations:** Department of Urology, Royal Surrey County Hospital NHS Trust, UK

**Keywords:** penile leiomyosarcoma, smooth muscle

## Abstract

Leiomyosarcomas of the penis are rare, with only 29 reported cases to date. We record the case of a patient who presented with a 2-year history of a seemingly indolent penile skin lesion. On histopathology of the local resection, a diagnosis of subcutaneous leiomyosarcoma was made. Specifically, leiomyosarcoma of the penile frenulum has not been clearly reported previously. The patient underwent a further excision to ensure an adequate resection margin and has had no disease recurrence at subsequent follow-up. Our case was of a lesion that, although clinically benign, was malignant and this possibility should be borne in mind when assessing patients.

**ABBREVIATIONS:** computerised tomography (CT), alpha-smooth muscle actin ($\alpha$-SMA), muscle-specific actin (HHF-35)

## INTRODUCTION

Leiomyosarcoma is the most common mesenchymal tumour in adults. However primary leiomyosarcomas of the penis are exceedingly rare.

## CASE REPORT

A 51-year-old gentleman presented with a 2-year history of a smooth 2-cm painless lesion on the skin of the penile frenulum. The lesion was excised under local anaesthetic as a day case.

Microscopic examination showed squamous epithelium with underlying subcutaneous tissue containing fascicles of spindle cells with intervening hyalinisation and oedema. The nuclei were markedly pleomorphic with a high, mitotic activity (Figs. 1 and 2). A diagnosis of Grade II subcutaneous leiomyosarcoma was made. Immunopositivity to desmin and quantification of MIB-1 nuclear positivity (Fig. 3) supported a diagnosis of leiomyosarcoma.

The excised lesion was found to abut the deep resection margin and so a further excision was undertaken with 5-mm resection margins, which showed no further tumour. Staging CT scan of the chest, abdomen, and pelvis was clear of metastatic disease and there has been no disease recurrence at 6-month follow-up.

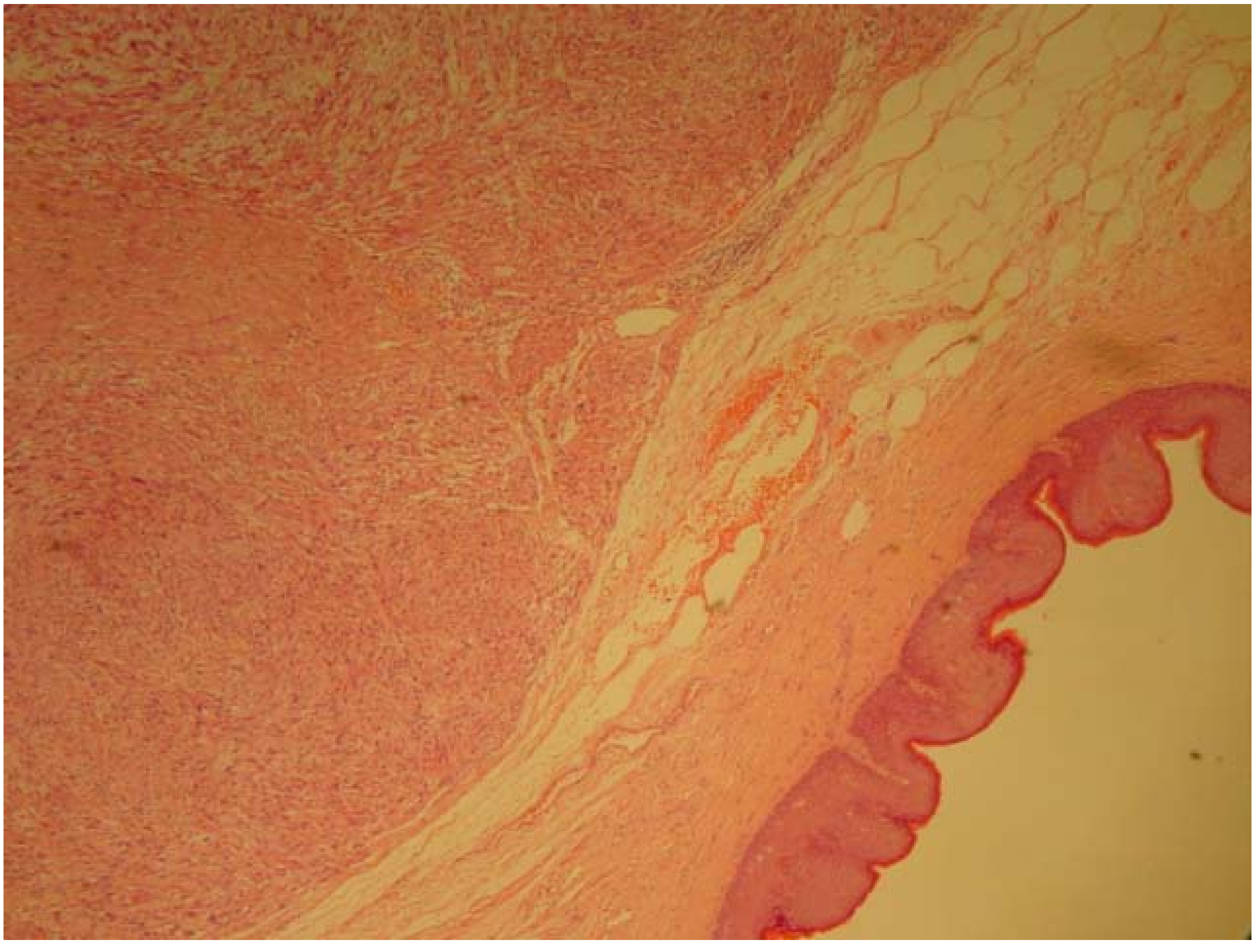

**FIGURE 1.** H + E stain of tissue section.

## DISCUSSION

Leiomyosarcoma of the frenulum is similar to cutaneous leiomyosarcoma arising from smooth muscle in the superficial tissues of the penile shaft. In contrast, deep penile leiomyosarcoma involve the corpus cavernosum and corpus spongiosum. Potential sources within the penis from which leiomyosarcoma can arise include: (1) the dartos muscle layer of the prepuce and shaft, (2) the arrector pili muscle associated with lanugo hairs on the penile shaft, (3) the muscular walls of superficial vessels situated outside the tunica albuginea, and (4) the muscular walls of the deep vascular complex that make up the corpus cavernosum and corpus spongiosum[1].

Macroscopically, the tumour is pink to grey in appearance and is either lobulated or plaque like[2]. Microscopically, immunohistochemical studies will generally confirm evidence of smooth muscle differentiation by showing strong cytoplasmic positivity for desmin, alpha-smooth muscle actin ($\alpha$-SMA), and muscle-specific actin (HHF-35)[3].

Clinically and pathologically, superficial tumours grow slowly and tend to recur locally, but have a higher salvage rate and a better prognosis. In comparison, deeper tumours exhibit a more aggressive behaviour with a tendency to invade the urethra causing urinary symptoms, to metastasize early, and are associated with a poorer prognosis. Despite radical surgery, the salvage rate is low and most patients die from metastatic disease within 2 years of diagnosis[4].

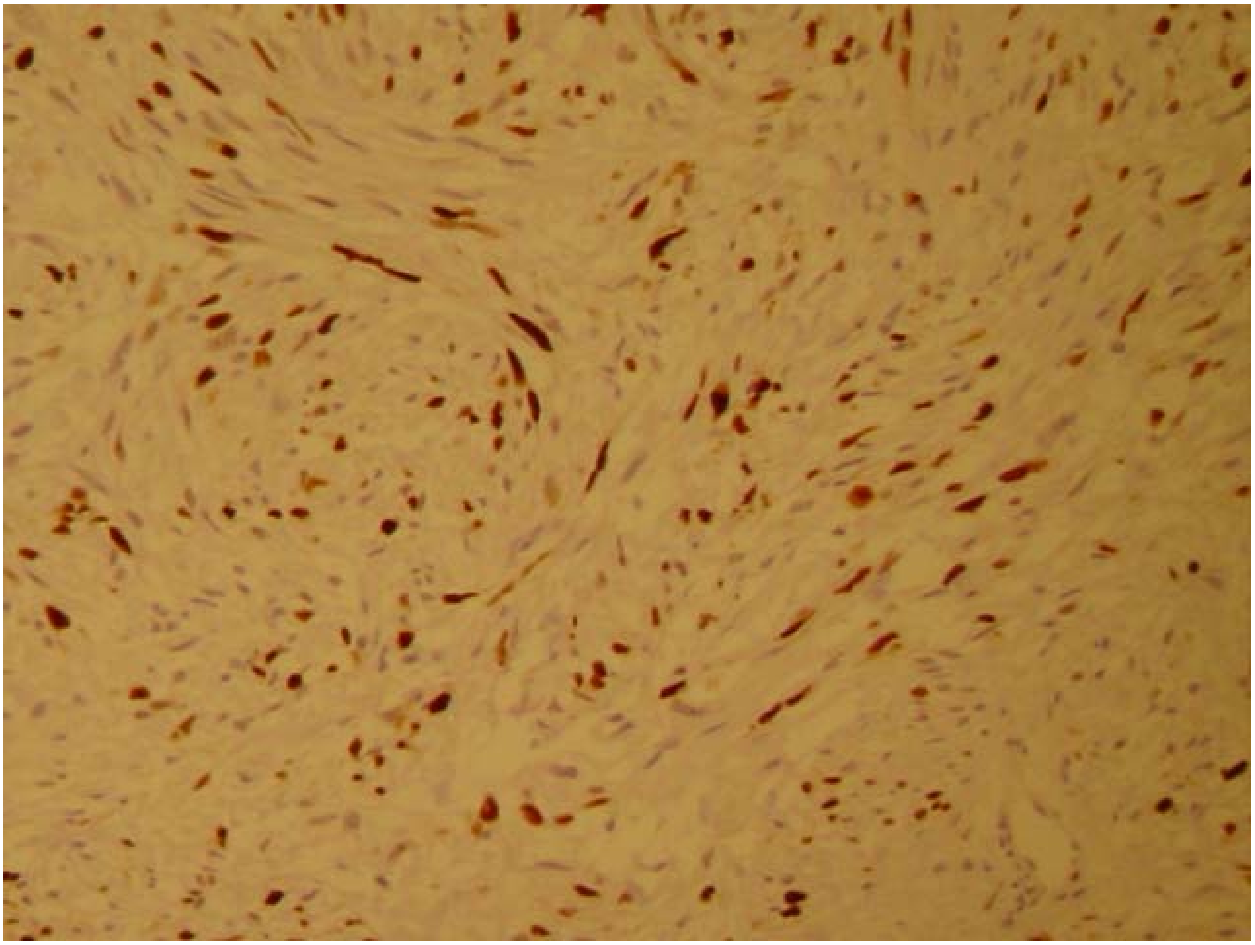

**FIGURE 2**. Higher power H + E stain, demonstrating fascicles of spindle cells, areas of hyalinisation, oedema, and a central mitotic figure.

Prognostic factors include tumour size, depth, and grade. This is classified as superficial or deep based in relation to the tunica albuginea. Tumour grade is reported to have a higher predictive value for metastases[5]. The modified French Federation of Cancer Centres Sarcoma Group Grading appears to be the most widely accepted grading system for soft tissue sarcomas. This is a three-tiered grading system based on the degree of differentiation, cellularity, number of mitoses, pleomorphism, and amount of necrosis.

Complete wide local excision with clear resection margins appears to be the optimal management for superficial tumours when small and well demarcated (<2 cm). Deeper tumours may also be amenable to wide local excision, however, if an adequate margin cannot be achieved by these means a partial or total penectomy may be required[6,7].

## CONCLUSION

Our case was of a lesion that, although clinically benign, was malignant and this possibility should be borne in mind when assessing patients.

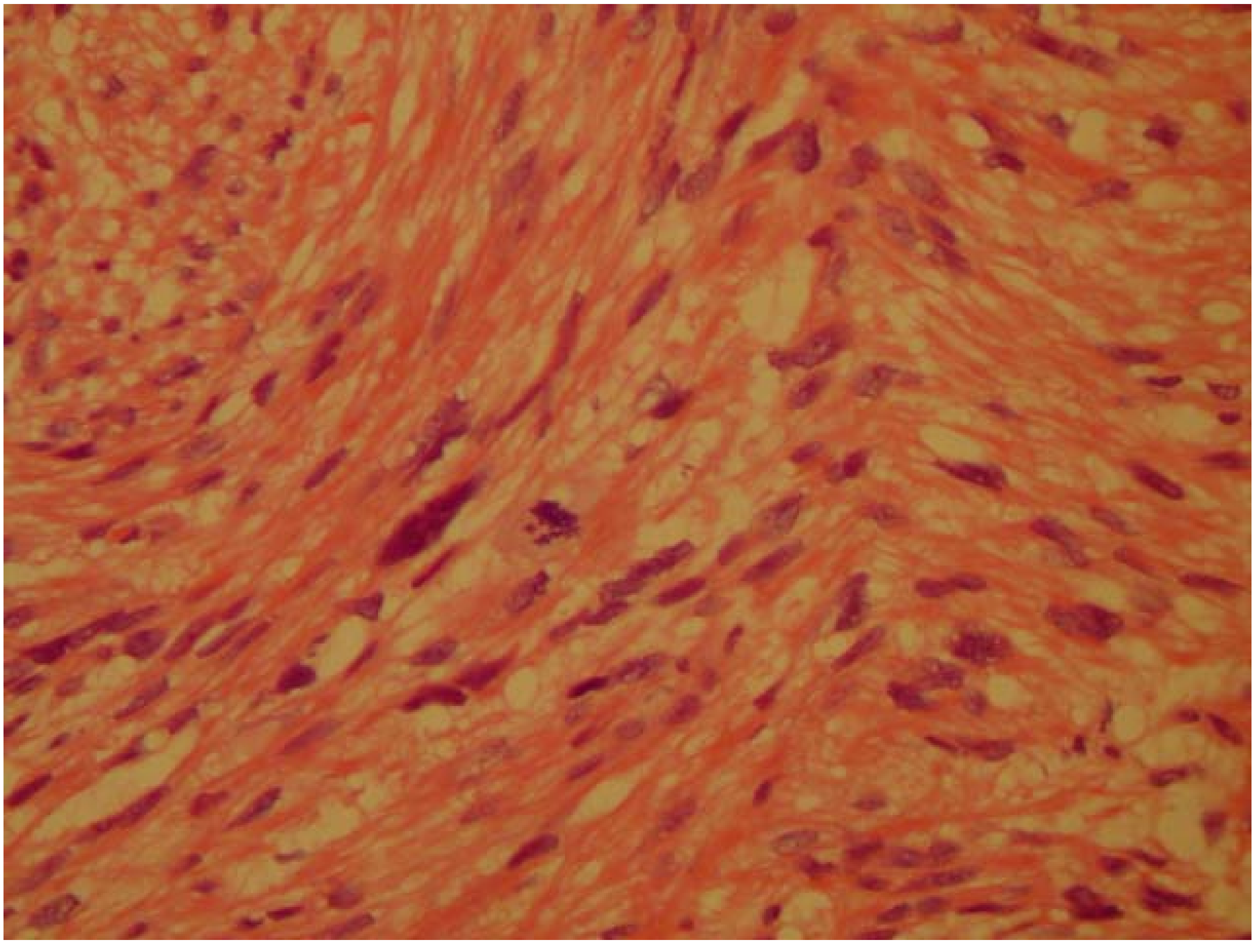

**FIGURE 3**. Immunohistochemistry MIB-1 stain of tissue section. MIB-1 is a cell nuclear antigen and a marker for proliferative activity. The stain shows the percentage of cells in cycle is 40% in this case, consistent with a malignant lesion.

## ACKNOWLEDGEMENTS

We thank Dr. Peter Jackson, Consultant Histopathologist (Royal Surrey County NHS) for his kind assistance in providing us with the relevant histopathology slides. We also thank Dr. Alex Freeman, Consultant Histopathologist (University College Hospital, London) and Mr. Alastair Henderson, Urology Specialist Registrar (Southwest Thames rotation) for their much-appreciated constructive comments.

## References

1. Fetsch, J.F., Davis, C.J., Jr., Miettinen, M., and Sesterhenn, I.A. (2001) Leiomyosarcoma of the penis. A clinicopathological study of 14 cases with review of the literature and discussion of the differential diagnosis. *Am. J. Pathol.* **28(1),** 115–125.
2. Dominici, A., Delle Rose, A., Stomaci, N., et al. (2004) A rare case of leiomyosarcoma of the penis with a reappraisal of the literature. *Int. J. Urol.* **11(6),** 440–444.

3. Sakamoto, A., Oda, Y., Yamamoto, H., et al. (2002) Calponin and h-caldesmon expression in atypical fibroxanthoma and superficial leiomyosarcoma. *Virchows Arch.* **440(4),** 404–409.
4. Isa, S.S., Almaraz, R., and Magovern, J. (1984) Leiomyosarcoma of the penis. Case report and review of the literature. *Cancer* **54(5),** 939–942.
5. Guillou, L., Coindre, J.M., Bonichon, F., et al. (1997) Comparative study of the National Cancer Institute and French Federation of Cancer Centers Sarcoma Group grading systems in a population of 410 adult patients with soft tissue sarcoma. *J. Clin. Oncol.* **15(1),** 350–362.
6. Pow-Sang, M.R. and Orihuela, E. (1994) Leiomyosarcoma of the penis. *J. Urol.* **151**, 1643–1645.
7. Mondaini, N., Palli, D., Saieva, C., et al. (2005) Clinical characteristics and overall survival in genitourinary sarcomas treated with curative intent: a multicentre trial. *Eur. Urol.* **47**, 468–473.
